# Online evaluation of the metabolic activity of *Ustilago maydis* on (poly)galacturonic acid

**DOI:** 10.1186/s13036-018-0128-1

**Published:** 2018-12-18

**Authors:** Markus Jan Müller, Sarah Stachurski, Peter Stoffels, Kerstin Schipper, Michael Feldbrügge, Jochen Büchs

**Affiliations:** 1 AVT – Biochemical Engineering, RWTH Aachen University, Jochen Büchs, Forckenbeckstr. 51, 52074 Aachen, Germany; 2 Institute for Microbiology, Cluster of Excellence on Plant Sciences, Heinrich-Heine University Düsseldorf, Universitätsstr. 1, 40225 Düsseldorf, Germany; 3 Bioeconomy Science Center (BioSC), 52426 Jülich, Germany

**Keywords:** Pectin degradation, *Ustilago maydis*, Oxygen transfer rate, Galacturonic acid, Exo-polygalacturonase, Enzymatic activity, Online measurement

## Abstract

**Background:**

Pectin is a rather complex and highly branched polysaccharide strengthening the plant cell wall. Thus, many different pectinases are required for an efficient microbial conversion of biomass waste streams with a high pectin content like citrus peel, apple pomace or sugar beet pulp. The screening and optimization of strains growing on pectic substrates requires both, quantification of the residual substrate and an accurate determination of the enzymatic activity. Galacturonic acid, the main sugar unit of pectin, is an uncommon substrate for microbial fermentations. Thus, growth and enzyme production of the applied strain has to be characterized in detail to understand the microbial system. An essential step to reach this goal is the development of online monitoring tools.

**Results:**

In this study, a method for the online determination of residual substrate was developed for the growth of the plant pathogenic fungus *Ustilago maydis* on pectic substrates such as galacturonic acid. To this end, an *U. maydis* strain was used that expressed a heterologous exo-polygalacturonase for growth on polygalacturonic acid. The growth behavior on galacturonic acid was analyzed by online measurement of the respiration activity. A method for the online prediction of the residual galacturonic acid concentration during the cultivation, based on the overall oxygen consumption, was developed and verified by offline sampling. This sensitive method was extended towards polygalacturonic acid, which is challenging to quantify via offline measurements. Finally, the enzymatic activity in the culture supernatant was calculated and the enzyme stability during the course of the cultivation was confirmed.

**Conclusion:**

The introduced method can reliably predict the residual (poly)galacturonic acid concentration based on the overall oxygen consumption. Based on this method, the enzymatic activity of the culture broth of an *U. maydis* strain expressing a heterologous exo-polygalacturonase could be calculated. It was demonstrated that the method is especially advantageous for determination of low enzymatic activities. In future, it will be applied to *U. maydis* strains in which the number of produced hydrolytic enzymes is increased for more efficient degradation.

**Electronic supplementary material:**

The online version of this article (10.1186/s13036-018-0128-1) contains supplementary material, which is available to authorized users.

## Background

The sugars required as a carbon source during the fermentation of agricultural waste streams usually originate from lignocellulosic biomass that is, e.g., thermochemically pretreated and enzymatically hydrolyzed [1–3]. The main fractions of all biomasses are cellulose, hemicellulose, lignin and pectin. However, the share of the fractions in the raw material depends on the origin of the biomass [4, 5]. For a complete breakdown of the raw material, numerous carbohydrate-active enzymes (CAZymes, [6]) for each component are required [7–9]. Since the commercial enzyme cocktails represent the main running cost of a biorefinery, the importance of screening for and microbial production of hydrolytic enzymes for biomass breakdown has increased in the last decades [8–11]. An important methodological challenge during the screening for new CAZyme producers is the reliable determination of residual polymeric substrate and of the enzymatic activity in the culture broth.

Strains of the family of *Ustilaginaceae* are promising candidates for the production of intrinsic or heterologous CAZymes [12, 13]. The best-characterized member of this family, *Ustilago maydis,* is a suitable workhorse in a biorefinery concept due to several reasons. The genome is fully sequenced and the methodology for genetic engineering is highly developed [14–17]. Furthermore, *U. maydis* has the capability of unicellular and non-filamentous growth with a high resistance to hydromechanical stress. This makes *U. maydis* favorable over established filamentous strains, as hydromechanical stress is usually elevated in stirred tank reactors [3, 18].

Originally, *U. maydis* was isolated as a phytopathogenic fungus, which provokes corn smut disease. During the nonpathogenic stage, *U. maydis* grows unicellular by budding with a haploid genome [19]. In response to plant cues, *U. maydis* cells fuse and grow as dikaryotic filaments which invade the maize tissue [15, 20, 21]. For efficient invasion, the fungus encodes a distinct set of hydrolytic enzymes that are required for traversing the plant cell wall [14, 22–24]. In addition, *U. maydis* produces diverse metabolites that can serve as valuable products in a consolidated bioprocess. Organic acids or glycolipids have been produced successfully in *U. maydis* [12, 13, 18, 25–28]. Overall, this organism can autonomously both, break down complex carbohydrate polymers and, simultaneously, convert the liberated sugars into high value products.

Beside containing cellulose, hemicellulose and lignin, specific biomass waste streams such as apple pomace, citrus peel or sugar beet pulp are also rich in pectin [1, 29, 30]. Pectin is a complex and branched heteropolysaccharide that reinforces the structure of plant cell walls [31, 32]. The most abundant polymer in pectin is homogalacturonan. It consists of linear, 1,4 α-d-linked galacturonic acid units that are partially methylated or acetylated [33, 34]. Polygalacturonic acid has a similar linear structure, but it lacks esterifications. For quantification of the released monomers, polysaccharides are usually hydrolyzed. In case of pectic polymers, the degradation by acid hydrolysis is unfavorable due to several reasons. Even harsh and elongated treatments do not completely degrade the pectic polymers [35]. Also, the hydrolysis rate strongly depends on pH, temperature and the degree of methylation of the hydrolyzed pectins [36]. Finally galacturonic acid monomers irreversibly form lactones under the conditions of hydrolysis what disables the subsequent analysis of the released sugars [37]. Thus, methods for complete degradation of pectic polymers usually combine physical and enzymatic hydrolysis with high-performance anion-exchange chromatography [38, 39]. To fully convert pectin-rich biomass waste streams enzymatically, a diverse set of hydrolytic enzymes is required [7, 40]. Few pectinolytic enzymes (including endo-polygalacturonase, pectin lyase and pectin methylesterase) have been annotated in the *U. maydis* genome [16, 24]. Polygalacturonic acid could act as a model substrate in this context as it shows, compared to homogalacturonan or pectin, a good accessibility for degradation into monomeric galacturonic acid by endo- or exo-polygalacturonases [41].

Several cellulase and xylanase genes have been shown to be present but silent in the yeast-like growth form of *U. maydis* [24]. Geiser *et al.* [22] successfully activated those enzymes by promoter exchange and demonstrated the capability of *U. maydis* to degrade certain plant cell wall components even in the unicellular growth form. Additionally, complementing the repertoire with heterologous enzymes was successful and resulted, e.g., in a strain that grows on polygalacturonic acid (Stoffels, Müller, *et al.* unpublished data). To this end, the codon-optimized gene for a heterologous exo-polygalacturonase PgaX originating from *Aspergillus tubingensis* was integrated into the *U. maydis* genome under the control of the strong promoter P_*oma*_ [42, 43]. This led to constitutive secretion of active exo-polygalacturonase for polygalacturonase degradation (Stoffels, Müller, *et al.* unpublished data). The main advantage of using the *U. maydis* expression system within this study was the unique presence of one CAZyme in the culture supernatant. This helped to gain a well-defined environment for the hydrolytic cleavage of polygalacturonic acid.

The Respiration Activity Monitoring System (RAMOS) enables the online monitoring of oxygen transfer rate (OTR), carbon dioxide transfer rate (CTR) and respiratory quotient (RQ) in eight parallel shake flask cultivations [44, 45]. This technique can be applied to monitor the metabolic activity of CAZyme producing organisms. The production of CAZymes enables the expressing organism to grow on the corresponding polymeric substrate. Thus, the metabolic activity of the organism grown on those polymeric substrates indicates the CAZyme activity. During cultivation, the overall oxygen consumption (OT) can be determined by integrating the OTR. Antonov *et al.* [46] recently analyzed the digestibility of different types of cellulose by online monitoring of the respiration activity of *T. reesei* Rut-C30. Alternative cellulase producers were subsequently investigated using this methodology, demonstrating its high potential [47]. The general concept of correlating metabolic activity during growth on a polymeric substrate with the enzymatic activity of the corresponding hydrolase was further investigated in this study for the activity of a heterologous exo-polygalacturonase acting on polygalacturonic acid.

## Results and Discussion

**Fig. 1:**
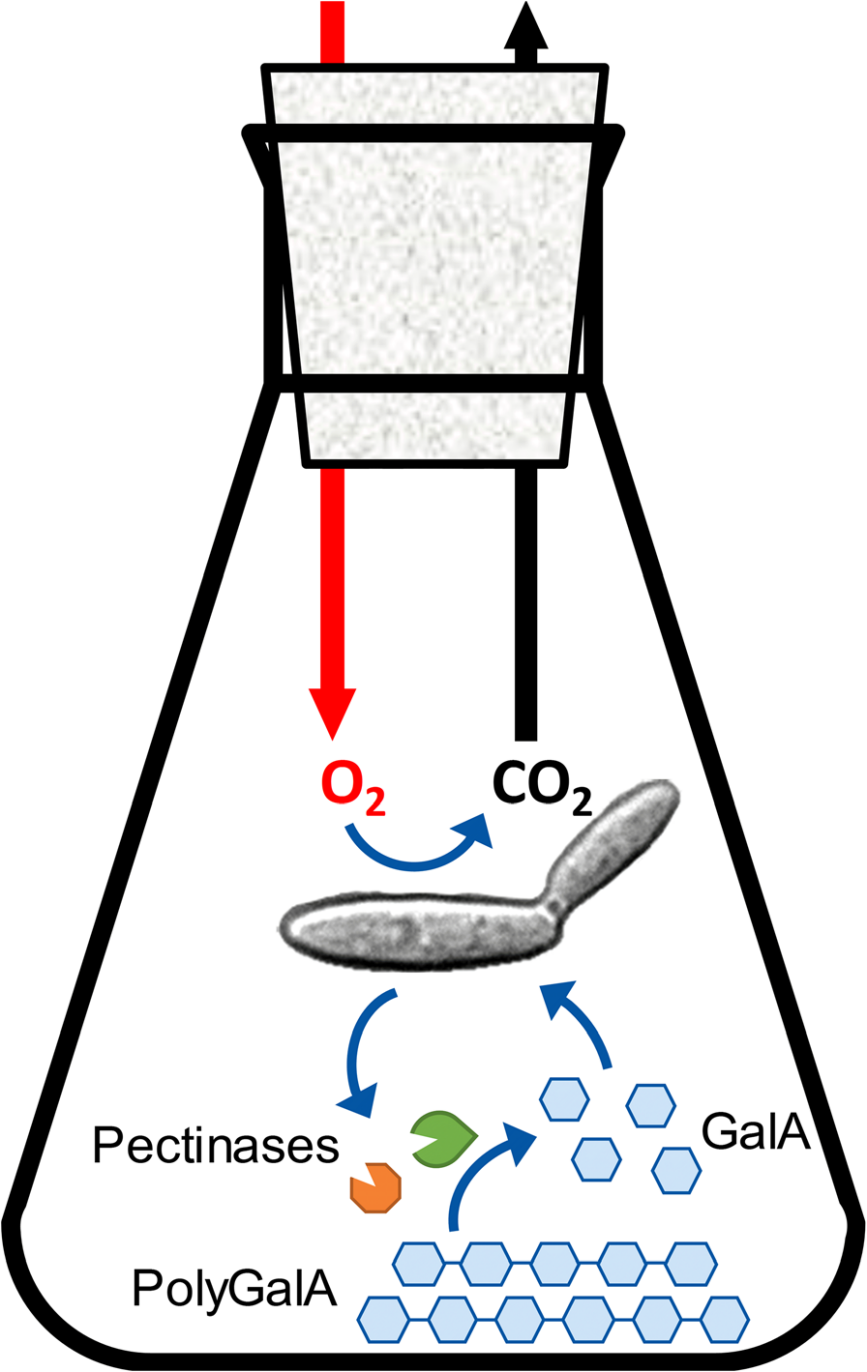
Process scheme of polygalacturonic acid (PolyGalA) degradation. *U. maydis* produces pectinases that degrade dissolved PolyGalA to monomeric galacturonic acid (GalA). GalA and oxygen are consumed in a fixed stoichiometric ratio while carbon dioxide is released. Oxygen consumption and carbon dioxide release are measured online. This concept enables the investigation of PolyGalA degradation by pectinolytic enzymes based on online monitoring of the metabolic activity

The applied measurement principle for growth of *U. maydis* on polygalacturonic acid is shown in Fig. 1. *U. maydis* is grown in RAMOS shake flasks with polygalacturonic acid as carbon source. If pectinases are available to degrade the polymeric substrate to monomers, monomeric galacturonic acid is consumed by *U. maydis*. The consumption of galacturonic acid correlates with the uptake of oxygen and the release of carbon dioxide in a specific molar ratio. Thus, by online measurement of the OTR and CTR, the pectinolytic activity of the produced enzymes in the culture broth can be determined. Furthermore, the relation between the amount of overall consumed oxygen and the residual substrate concentration in the culture supernatant was investigated. The resulting method for prediction of the residual substrate concentration was applied for monomeric galacturonic acid but, more importantly, also for polygalacturonic acid that cannot be quantified easily by offline measurement.

### Online monitoring of *U. maydis* growth on galacturonic acid

As first step, the growth of the protease deficient strain *U. maydis* AB33P5ΔR on galacturonic acid was characterized by online monitoring of the metabolic activity. AB33 is known to grow in a stable unicellular state making it applicable up to fermenter scale [48]. Furthermore, the strain produces less lipids compared to other *U. maydis* strains like MB215 [49]. This provides the potential to achieve high carbon fluxes towards a desired product. The specific quintuple protease deletion strain, previously generated by Sarkari *et al.* [50], was chosen to gain a higher enzyme stability in the culture supernatant.

The mineral medium described by Geiser *et al.* [22] was adapted for the cultivation on pectic substrates. Nitrogen limitation is known to induce itaconic acid production in certain *U. maydis* strains, but significantly reduces growth [3, 51–53]. As this study focuses on the growth on complex substrates, the ammonium chloride concentration was elevated from 0.8 to 4.0 g/L to prevent a secondary substrate limitation. The vitamin solution was omitted, as no advantageous effect was visible during the cultivation (see Additional file 1). Low amounts of glucose have previously been used to achieve an exponential growth of the culture before entering the second growth phase on the main carbon source [46]. Thereby, the overall cultivation time is decreased significantly. A reference cultivation of *U. maydis* demonstrating the slow growth on galacturonic acid without addition of glucose is shown in Additional file 2. Cultivation on pure galacturonic acid also results in lower measured OTR values. This is unfavorable as the signal-to-noise ratio decreases resulting in a lower data quality. Therefore, 4 g/L glucose and 20 g/L galacturonic acid were used as carbon sources. The pH value was expected to increase when *U. maydis* consumes an acidic carbon source. To prevent a strong pH shift towards basic values, the originally reported 0.1 M MES buffer was replaced with 0.2 M MOPS buffer. MOPS buffer (pKa = 7.2) provides a larger buffer capacity towards higher pH values compared to MES buffer (pKa = 6.1).

**Fig. 2:**
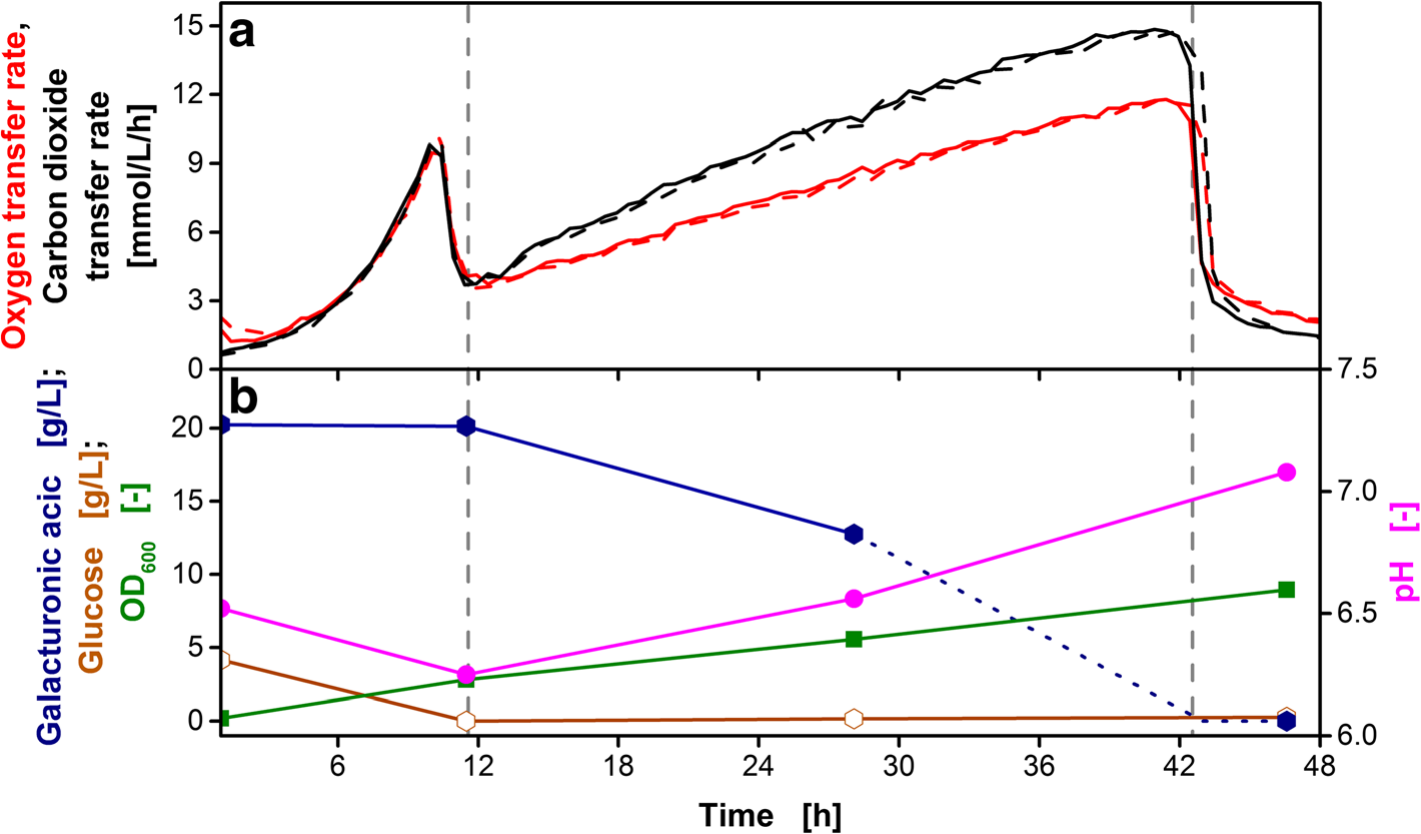
Cultivation of *U. maydis* AB33P5ΔR on galacturonic acid. The strain was grown on glucose (4 g/L) and galacturonic acid (20 g/L) as carbon sources. Vertical grey dashed lines indicate depletion of glucose (11.5 h) and galacturonic acid (43 h). **a** Biological duplicates of oxygen transfer rate and carbon dioxide transfer rate, represented each as line and dashed line. **b** Concentrations of galacturonic acid and glucose, OD_600_ and pH. The blue dotted line represents the assumed course of galacturonic acid concentration since the OTR indicates galacturonic acid depletion after 43 h. Culture conditions: modified Verduyn medium, 0.2 M MOPS, initial pH 6.5, 250 mL flask, filling volume 20 mL, shaking frequency 300 rpm, shaking diameter 50 mm, initial OD_600_ = 0.2, T = 30 °C

Figure 2a shows the OTR and CTR over time of an *U. maydis* AB33P5ΔR cultivation in the modified mineral medium containing glucose (4 g/L) and galacturonic acid (20 g/L) as carbon sources. In each experiment, biological duplicates of the cultivation conditions were investigated. The variation between the duplicates was very low, indicating a high reproducibility. The first exponential growth phase of *U. maydis* AB33P5ΔR was characterized by consumption of glucose as preferred carbon source. The maximal OTR of 10 mmol/L/h was reached after 10.5 h. With the depletion of glucose, the OTR decreased to 3.5 mmol/L/h at 11.5 h, indicating a metabolic adaption of the organisms for the second growth phase. Depletion of glucose was also verified by offline sampling as depicted in Fig. 2b (first vertical grey dashed line). During the first growth phase, no galacturonic acid was consumed. The optical density increased from 0.2 to 2.8. The pH slightly decreased from 6.52 to 6.25 due to ammonia consumption [54, 55].

During the second growth phase, the culture consumed galacturonic acid until substrate depletion after 43 h (second vertical grey dashed line). The OTR increased at a lower rate when compared to growth on glucose, indicating a poorer metabolization rate of galacturonic acid. This slow growth on galacturonic acid is characteristic for *U. maydis* and these results are consistent with previous reports [23]. The OTR increased towards a maximal OTR of 11.8 mmol/L/h after 41 h. The finally measured optical density was 9.0. The end of the cultivation was characterized through a steep descent of the OTR after 43 h. At this point, all galacturonic acid was consumed. The pH increased during the galacturonic acid consumption phase to 7.1. This behavior is typical during the consumption of acidic carbon sources as they cross the cell membrane only in their protonated form [56]. At the end of the cultivation, 1.45 g/L ammonium chloride remained in the culture supernatant, indicating that the elevated concentration of 4 g/L ammonium chloride was sufficient to prevent nitrogen limitation.

The RQ describes the ratio of CTR to OTR. It is known as a central parameter to characterize the metabolic activity of microorganisms during growth on multiple substrates of different degree of reduction [45]. Additional file 3b shows the RQ over time for the cultivation on galacturonic acid. During growth on glucose, OTR and CTR are identical, which results in a RQ of about 1. Similar RQ values were obtained by others for the growth of *U. maydis* and yeasts while growing on glucose [3, 45]. During the growth on galacturonic acid, the CTR values are higher than the OTR, resulting in an RQ of 1.3 to 1.4. Finally, after depletion of galacturonic acid, the RQ drops below 1, indicating that for maintenance internal reduced storage compounds like, e.g., lipids are consumed.

To understand the different distinct levels of the RQ, a carbon balance was developed for the cultivation on glucose and galacturonic acid. Assuming pure biological combustion of the substrate for energy metabolism, the substrate is oxidized to carbon dioxide and water as described by Eq. (1).

1 $$ {\nu}_S{C}_6{H}_{10}{O}_7+{\nu}_O{O}_2\rightarrow {\nu}_CC{O}_2+{\nu}_H{H}_2O $$

*ν*: stoichiometric coefficients for substrate galacturonic acid (S), oxygen (O), carbon dioxide (C) and water (H)

The corresponding theoretical RQ value (RQ_theo_) can be calculated from the stoichiometric coefficients as described by Eq. (2). They are 1.0 and 1.2 for glucose and galacturonic acid respectively as listed in Table 1.

2 $$ R{Q}_{theo}=\frac{\nu_C}{\nu_O} $$

**Table 1:** Stoichiometric coefficients and respiratory quotient for carbon balance

Conditions	C-source	Sum formula	ν_S_	*ν*_N_	*ν*_O_	*ν*_X_	*ν*_C_	*ν*_H_	RQ_theo_	*Y*_*X*/ *S*_
Pure combustion	Glc	C_6_H_12_O_6_	1	-	6	-	6	6	1.00	-
Pure combustion	GalA	C_6_H_10_O_7_	1	-	5	-	6	5	1.20	-
Combustion and biomass formation	Glc	C_6_H_12_O_6_	1	0.53	2.13	3.65	2.35	3.46	1.10	0.51
Combustion and biomass formation	GalA	C_6_H_10_O_7_	1	0.29	2.92	1.97	4.06	3.63	1.38	0.27

RQ_theo_: theoretical respiratory quotient [-]

However, to represent conditions that are more realistic, Eq. (1) has to be extended with the formation of biomass and the consumption of ammonia as described in Eq. (3).

3 $$ {\nu}_S{C}_6{H}_{10}{O}_7+{\nu}_NN{H}_3+{\nu}_O{O}_2\rightarrow {\nu}_XX+{\nu}_CC{O}_2+{\nu}_H{H}_2O $$

*ν*: stoichiometric coefficients for ammonia (N) and biomass (X)

Balancing of Eq. (3) requires the stoichiometric coefficient ν_X_. The elemental composition of *U. maydis* under N-unlimited conditions was previously determined to be CH_1.826_O_0.579_N_0.145_ [3]. Based on the determination of the cell dry weight and measurement of the residual substrate concentration by HPLC, a yield coefficient for biomass formation (Y_X/S_) of 0.51 and 0.27 g_X_/g_S_ was calculated according to Eq. (4) for glucose and galacturonic acid, respectively. The results are listed in Table 1.

4 $$ {Y}_{XS}=\frac{\varDelta X}{\varDelta S} $$

Y_XS_: yield coefficient [g_X_/g_S_]

ΔX, ΔS: change of biomass (X) or substrate (S) concentrations between sampling points [g/L]

Y_XS_ was used to define ν_X_ as described in Eq. (5).

5 $$ {\nu}_X={Y}_{XS}\ \frac{M{W}_X}{M{W}_S} $$

MW_X_, MW_S_: molecular weight of biomass (X) or substrate (S) [g/mol]

Using the experimental value for ν_X_ in Eq. (3) results in stoichiometric coefficients and the RQ_theo_ values for combined combustion and biomass formation as given in Table 1. The calculated RQ_theo_ values of 1.10 and 1.38 for glucose and galacturonic acid, respectively, matched the experimentally determined data (Additional file 3b). This indicates that no relevant carbon fluxes other than combustion to carbon dioxide for energy generation and cell growth occur. Thus, the carbon balance is closed for the cultivation on glucose and galacturonic acid.

### Correlation of oxygen uptake with consumed galacturonic acid

One aim of this study is to provide a method for predicting the residual concentration of galacturonic acid from the OT during the galacturonic acid consumption phase. The correlation developed in this section will later be applied on the growth of *U. maydis* AB33P5ΔR/AtPgaX on polygalacturonic acid. For consistence with the later application, this strain was already used for the development of the correlation introduced within this chapter.

**Fig. 3:**
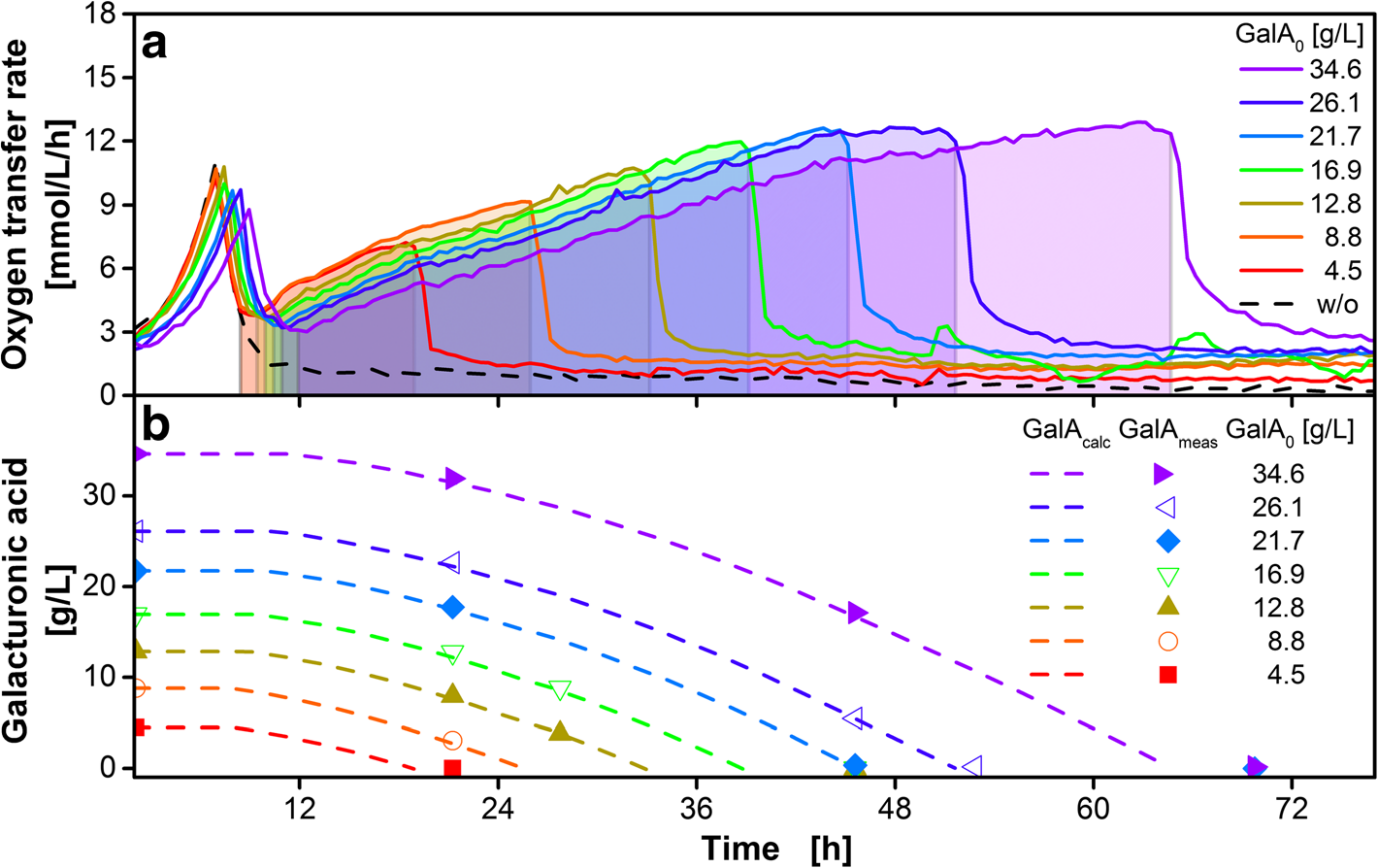
Cultivation of *U. maydis* AB33P5ΔR/AtPgaX with varying galacturonic acid and buffer concentrations. The medium was supplemented with glucose (4 g/L) and varying amounts of initial galacturonic acid (GalA_0_) and MOPS buffer. 0, 4.5, 8.8, 12.8, 16.9, 21.7, 26.1 and 34.6 g/L GalA_0_ was supplemented with 0.1, 0.1, 0.1, 0.15, 0.2, 0.25, 0.3 and 0.4 M MOPS, respectively. **a** Oxygen transfer rate. The galacturonic acid consumption phase is indicated by the shaded area below the OTR curve for each cultivation. **b** Dashed lines represent the predicted residual substrate concentration in the culture medium (GalA_calc_) according to Eq. (6). Symbols represent offline measured values (GalA_meas_). Culture conditions: modified Verduyn medium, initial pH 6.5, 250 mL flask, filling volume 20 mL, shaking frequency 300 rpm, shaking diameter 50 mm, initial OD_600_ = 0.65, T = 30 °C

For experimentally developing a correlation of the OT with the galacturonic acid supplemented in the medium, the concentration of galacturonic acid was varied from 0 to 34.6 g/L. As the pH is increasing during the consumption of galacturonic acid, the buffer concentration was increased in proportion to the substrate concentrations with a minimal buffer concentration of 0.1 M. Figure 3a shows the OTR over time of *U. maydis* AB33P5ΔR/AtPgaX in media containing glucose (4 g/L) and varied concentrations of both, galacturonic acid (0 to 34.6 g/L) and MOPS buffer (0.1 to 0.4 M). Compared to Fig. 2a, the same two growth phases on glucose (lasting for 10 to 12 h) and galacturonic acid can be distinguished. The growth on glucose was only slightly slowed down at elevated galacturonic acid and buffer concentrations due to increased osmolarity. In line with this, previous studies reported the high robustness of *U. maydis* under elevated osmolarities while not remaining totally unaffected [3, 57]. As expected, the OTR of the control cultivation without galacturonic acid dropped down to a basal level already after 10 h. All other cultivations showed the typical two-stage growth behavior. Galacturonic acid was depleted after different cultivation times, according to the initially supplemented substrate concentration. The corresponding OTs for the galacturonic acid consumption phases are indicated in Fig. 3a by the shaded areas under the curves.

**Fig. 4:**
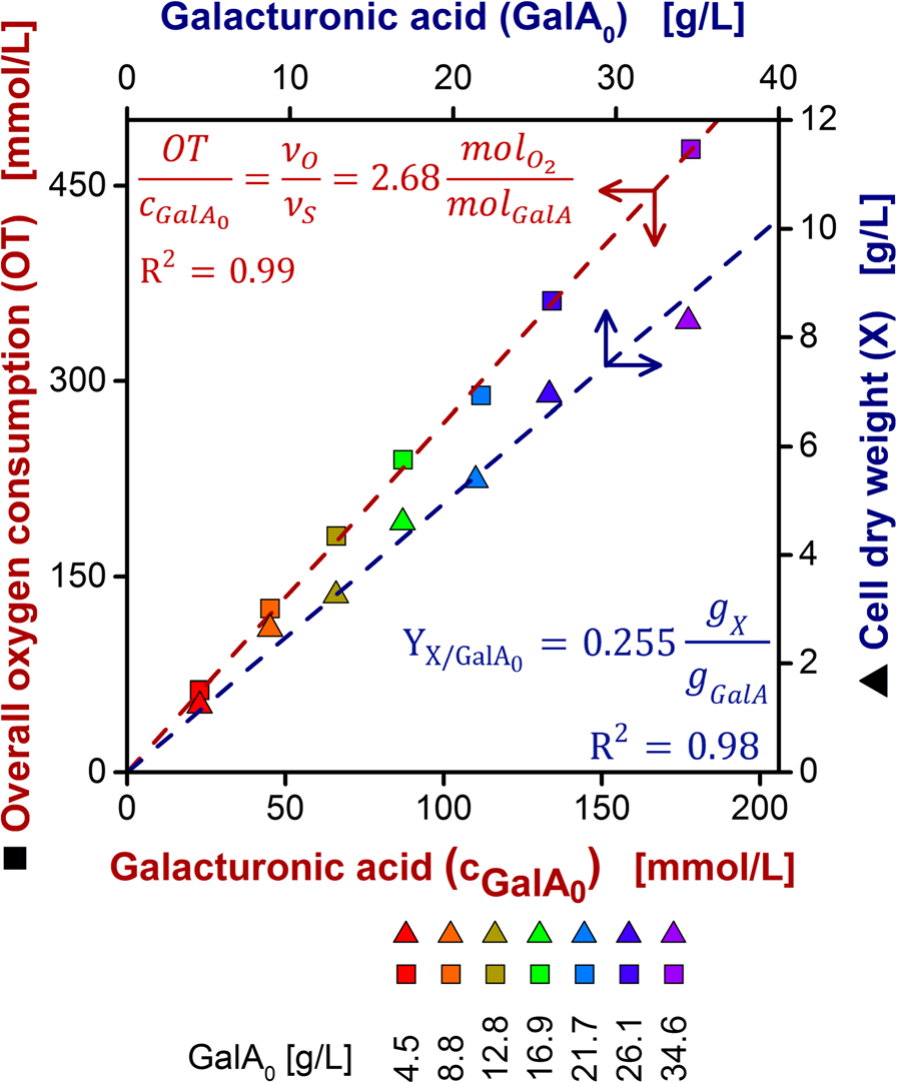
Correlation of overall oxygen consumption (OT, squares, red axes) and produced biomass (X, triangles, blue axes) to initial galacturonic acid concentration (${c}_{Gal{A}_0}$ and *GalA*_0_ respectively). The colored data points correspond to the equally colored cultivations shown in Fig. 3. The galacturonic acid concentrations were determined via offline measurement. The OT is taken into account for the galacturonic acid consumption phase. A linear fit was plotted (dashed lines, arrows indicate corresponding axes). The reciprocal value of $\frac{\nu_O}{\nu_S}$ can be substituted in Eq. (6). The calibration factors and coefficients of determination are specified in the plot

In Fig. 4 (squares), the OT during the galacturonic acid consumption phase (see also shaded areas in Fig. 3a) was plotted against the initial galacturonic acid concentration. The linear regression of the data points (red dashed line) demonstrates the excellent correlation of the two parameters. Thus, the reciprocal value of the experimental calibration factor $\frac{\nu_O}{\nu_S}$ can be used to predict the residual substrate concentration GalA_calc_ during the entire cultivation according to Eq. (6).

6 $$ (Poly){GalA}_{calc}=M{W}_{GalA}\left({c}_{(Poly) Gal{A}_0}-\frac{\nu_S}{\nu_O} OT\right) $$

(Poly)GalA_calc_: calculated residual (poly)galacturonic acid concentration [g/L]

MW_GalA_: molecular weight of (poly)galacturonic acid [g/mol]

${c}_{(Poly) Gal{A}_0}$: initial (poly)galacturonic acid concentration [mol/L]

OT: overall oxygen consumption during (poly)galacturonic acid consumption phase [mol/L]

GalA_calc_ is shown for all cultivations in Fig. 3b (dashed lines). The predicted GalA_calc_ is in very good agreement with offline measured concentrations of galacturonic acid (GalA_meas,_ Fig. 3b, symbols). This verifies the high reliability of the predicted concentrations. Furthermore, the predicted depletion of substrate correlates well with the sharp drop in the OTR.

The carbon balancing introduced in the previous chapter requires experimental values for Y_X/S_. Online monitoring of the metabolic activity (OTR) enabled sampling right after glucose was depleted and shortly after depletion of galacturonic acid. Fig. 4 (triangles) shows the data that was used to calculate the yield coefficient Y_X/S_ as described by Eq. (4). As expected, the cell dry weight increases with increasing galacturonic acid concentrations as more biomass is formed. The good agreement of the data points with a linear fit indicates a cultivation that is not limited by other substrates than the carbon source. Compared to the yield coefficient on glucose, determined from the first growth phase with Y_X/S_ = 0.51 g_X_/g_S_, *U. maydis* does produce less biomass from galacturonic acid (Y_X/S_ = 0.27 g_X_/g_S_). This can partially be explained by the degree of reduction as a parameter for the energy content of the substrate. For galacturonic acid, the degree of reduction is 3.33 per carbon atom. Compared to that, glucose shows a degree of reduction of 4 per carbon atom. Thus, the energy content of galacturonic acid is lower than of glucose. This is also in accordance with the lower growth rate on galacturonic acid compared to glucose, as the increase of the OTR during growth on galacturonic acid is not exponential (Fig. 2a) [23].

Comparing the different OTR profiles in Fig. 3a, it should be noted that the increase in the OTR becomes less steep for the cultivation with 34.6 g/L galacturonic acid towards the end of the cultivation. This might be associated with a slight shift in the cultivation pH (final pH: 7.3, see also Additional file 4). *U. maydis* is usually cultivated at pH values of 5.5 to 6.5 [13]. Cultivations above that optimal range can reduce the specific growth rate.

### Characterization of growth on polygalacturonic acid

To achieve metabolization of polygalacturonic acid, strain AB33 P5ΔR/AtPgaX was applied. Two reference cultivations of this strain are shown in Additional file 5. In one cultivation, 20 g/L glucose was added as single carbon source (black) while in the other cultivation 20 g/L polygalacturonic acid without supplemented glucose was provided (green). No growth was observed for the cultivation with pure polygalacturonic acid, indicating that polygalacturonic acid is not sufficient as a sole carbon source. This behavior is contradictory to previous reports of the yeast-like growing AB33 progenitor strain FB2 that was able to metabolize polygalacturonic acid [23]. Other reports clearly attribute CAZyme expression to the plant invasive, filamentous growth form that was not part of this study [24, 41]. Differences between the studies might occur from different medium composition, substrate suppliers or cultivation conditions and are currently under investigation. As the strain grows exponentially on glucose, the medium containing polygalacturonic acid was supplemented with 4 g/L glucose as a starter carbon source. Such supplementation of the culture medium with 4 g/L glucose was already used in the previous section for the cultivation on monomeric galacturonic acid to decrease the overall cultivation time by an increased cell density at the beginning of the second growth phase. Glucose as a starter carbon source plays a much more important role for cultivations on polygalacturonic acid. In this case, glucose activates the regulatory elements for production and secretion of hydrolytic enzymes. Previous studies on the implemented P_*oma*_ promoter indicate a strong influence of the applied carbon source on the expression of the target protein [20]. The strongest expression was achieved when glucose, fructose, sucrose or arabinose were provided as carbon source while significantly lower expression rates were achieved on xylose, maltose or glycerol [20]. Assuming a similar decreased activity of the P_*oma*_ promoter on (poly)galacturonic acid could explain the lack of metabolic activity for the *U. maydis* cultivation on polygalacturonic acid as sole carbon source.

**Fig. 5:**
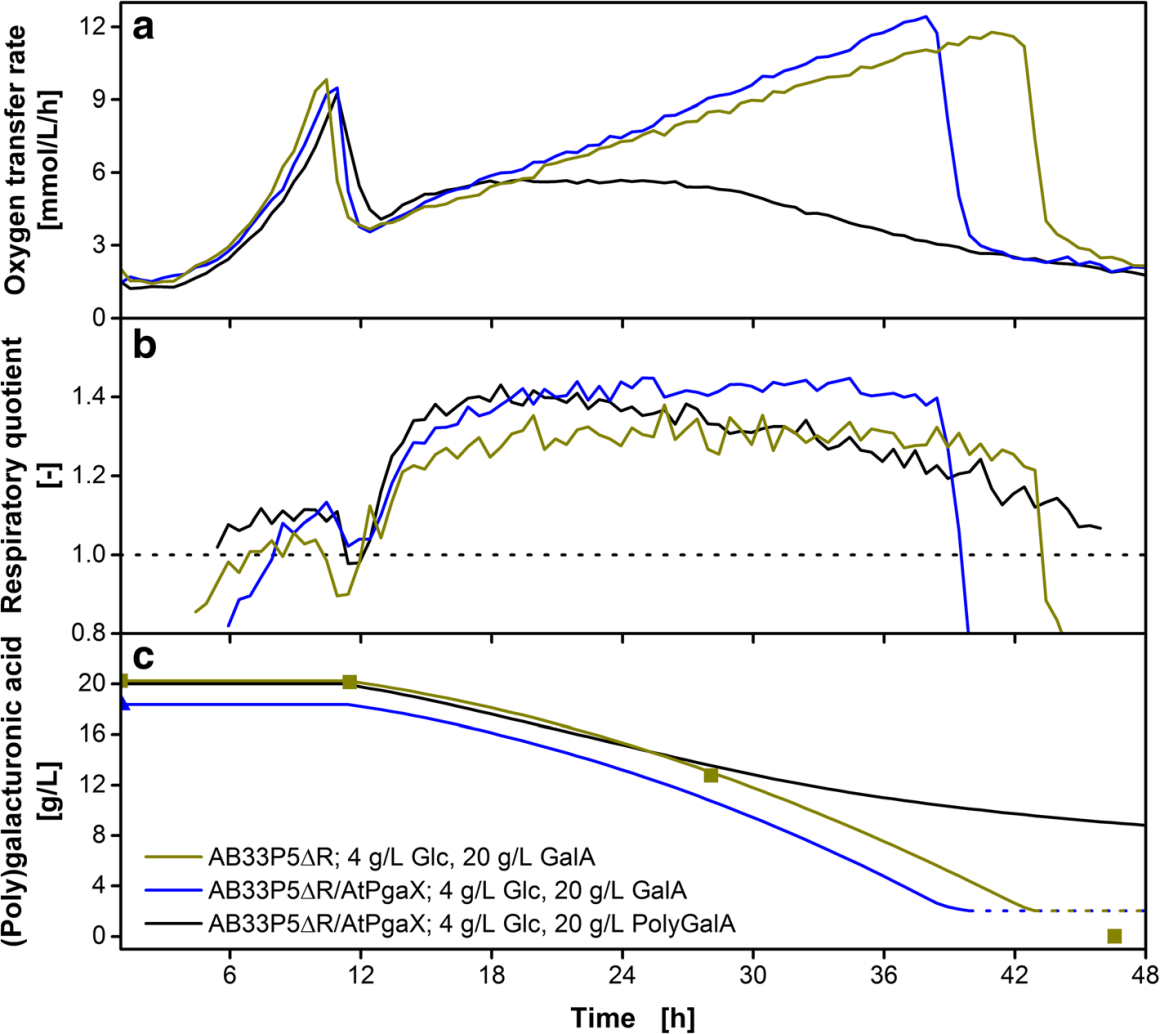
Cultivation of *U. maydis* AB33P5ΔR and AB33P5ΔR/AtPgaX on (poly)galacturonic acid. The strains were grown on glucose (4 g/L) and galacturonic acid or polygalacturonic acid (20 g/L) as carbon sources. **a** Mean value of duplicates of oxygen transfer rate. **b** Mean value of duplicates of respiratory quotient. The dotted horizontal line represents RQ = 1. **c** Predicted residual substrate concentration in the culture medium (GalA_calc_ or PolyGalA_calc_) according to Eq. (6). The RQ drops below 1 after 40 and 44 h, respectively, indicating depletion of galacturonic acid. Thus, the line for GalA_calc_ in Fig. 5c is dotted from that time point on. Symbols represent offline-measured values. The mean values of biological duplicates are shown for online measurements for better clarity. Culture conditions: modified Verduyn medium, 0.2 M MOPS, initial pH 6.5, 250 mL flask, filling volume 20 mL, shaking frequency 300 rpm, shaking diameter 50 mm, initial OD_600_ = 0.2, temperature 30 °C

To determine the ability of the new strain to grow on monomeric galacturonic acid, a second reference cultivation on glucose and galacturonic acid was conducted. Figure 5a shows the OTR over time for the cultivation of AB33P5ΔR/AtPgaX (blue) and its progenitor strain AB33P5ΔR (olive green) on 4 g/L glucose and 20 g/L monomeric galacturonic acid. Both strains showed a similar pattern of the OTR course demonstrating their comparability during the cultivation. In contrast to AB33P5ΔR lacking the exo-polygalacturonase, AB33P5ΔR/AtPgaX also showed metabolic activity when cultivated on polygalacturonic acid (black). However, in comparison to the cultivation on monomeric substrate, the cultivation on polygalacturonic acid behaved differently. Similar OTR patterns were previously observed for cultivations of *T. reesei* on cellulose and divided into three different phases [46, 47]. The cultivation of AB33P5ΔR/AtPgaX on polygalacturonic acid could be divided into the same phases. The first phase during galacturonic acid consumption (13 – 18 h) could be described by an identical OTR for both cultivations, with polygalacturonic acid (black) and monomeric galacturonic acid (blue) as main carbon source. This indicates an unlimited growth on the monomer in both cases. The corresponding RQ (Fig. 5b) showed a characteristic pattern with an RQ of 1.1 during the glucose consumption phase (< 10 h), a short kink after glucose depletion (10 to 12 h) and an increase to 1.3 -1.4 at the beginning of the galacturonic acid consumption phase (< 12 h). After 18 h, the OTR of the cultivation on polygalacturonic acid entered the second phase. A plateau of OTR = 5.7 mmol/L/h was reached (18 to 26 h). This indicates a substrate limitation. The current enzymatic activity in the culture supernatant led to a constant liberation rate of galacturonic acid monomers. The RQ remained on a high level of 1.3 – 1.4, verifying that all hydrolyzed sugar units are immediately consumed. Using the method for prediction of the residual substrate concentration described by Eq. (6) enabled the calculation of the polygalacturonic acid concentration (PolyGalA_calc_). In this case, the molar concentration refers to the monomeric galacturonic acid without considering the degree of polymerization. As the OTR of the cultivation shown in Fig. 5a (black curve) remained constant, no new exo-polygalacturonase is produced by the strain. This plateau in the OTR correlates with the linear decrease of PolyGalA_calc_ depicted in Fig. 5c (black curve). Thus, during this phase, the culture is limited due to enzymatic activity in the supernatant, meaning that a higher enzymatic activity would lead to a higher OTR. After 26 h, the cultivation on polygalacturonic acid entered a third phase. The OTR starts to decrease towards a basal level. After 42 h, the OTR of both cultures, with polygalacturonic and monomeric galacturonic acid, ran in parallel. Previously, a decreasing OTR during the cultivation of *T. reesei* on cellulose was annotated with a decreased accessibility of the substrate [46]. In contrast to crystalline suspended cellulose particles, polygalacturonic acid is solubilized in the medium. Therefore, physical inaccessibility could not be the reason for the decreasing OTR in the last phase (26 to 42 h) and the incomplete substrate utilization. Looking at the overall consumed polygalacturonic acid at the end of the cultivation (Fig. 5c, black line), only half of the supplemented substrate was used. The decreasing OTR, thus, describes a decreasing liberation rate of galacturonic acid even under high residual polygalacturonic acid concentrations. The applied exo-polygalacturonase is highly specific towards unesterified polygalacturonic acid [43, 58, 59]. Polygalacturonic acid is not polymerized *de novo* but originates from processed pectin or homogalacturonan. This material is likely to contain remaining esterifications, pectic side chains or rhamnose units in the backbone. As the exo-polygalacturonase is acting specifically on the non-reducing end of the polymer chain, a single unusual polymer unit blocks the hydrolysis of the entire polymer chain at its reducing end. Overall, this effect leads to the high fraction of remaining substrate in the culture. In line with a decreasing galacturonic acid consumption rate, the RQ deceased during this third phase towards the level of pure combustion indicating a decreasing biomass formation and increasing cell maintenance. During this last phase, the cultivation could be characterized as substrate limited. In this case, more substrate would lead to a higher OTR.

During the second phase of limitation by the enzymatic activity, the OTR level during the linear decrease of PolyGalA_calc_ could be applied as a read-out for online determination of the enzymatic activity. The constant plateau in the OTR indicates the enzymatic hydrolysis as growth-limiting parameter of the cultivation. All liberated monomeric galacturonic acid is immediately consumed. Eq. (7) describes the calculation of the enzymatic activity of the culture supernatant from the plateau level OTR*. One unit of enzyme activity is defined as the amount of enzyme required to liberate 1 μmol of galacturonic acid per minute.

7 $$ E= OT{R}^{\ast}\frac{\nu_S}{\nu_O} $$

E: enzymatic activity [U/mL]

OTR^*^: oxygen transfer rate at plateau [μmol/mL/min]

The determined enzymatic activity in the culture supernatant was 35.1 mU/mL. This method has several advantages over offline enzymatic assays. It is possible to measure even low activities that are hard to detect via offline enzymatic assays. Additionally, the method is not affected by possible product inhibition of the enzyme, a known issue for several pectinases or cellulases [60, 61]. Finally, the determination is conducted under cultivation conditions and, thus, more reliable with regard to a later process scale-up compared to artificial conditions in offline enzyme assays.

### Influence of initial glucose concentration on enzyme expression

**Fig. 6:**
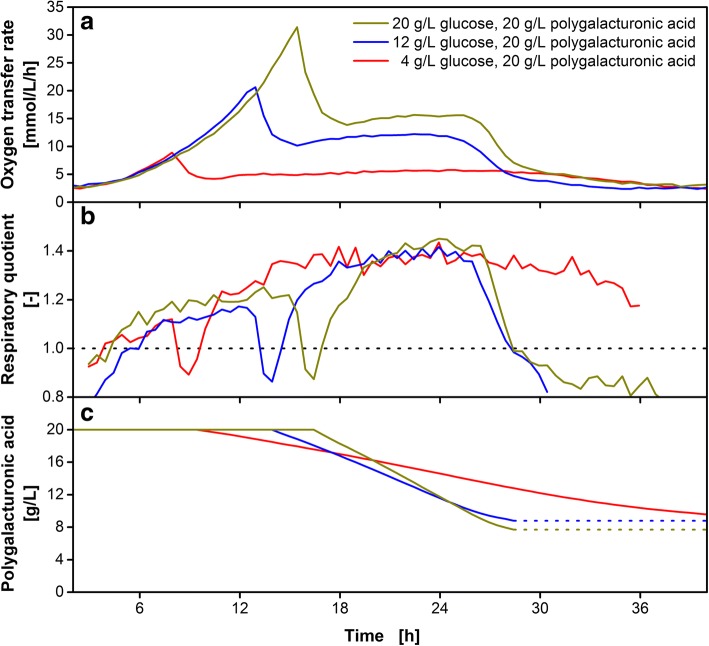
Cultivation of *U. maydis* AB33P5ΔR/AtPgaX on polygalacturonic acid with varying initial glucose concentrations. The strain was grown on glucose (4, 12 and 20 g/L) and polygalacturonic acid (20 g/L) as carbon sources. **a** Mean value of biological duplicates of oxygen transfer rate. **b** Mean value of biological duplicates of respiratory quotient. The dotted horizontal line represents RQ = 1. **c** Predicted residual polygalacturonic acid concentration in the medium (PolyGalA_calc_) according to Eq. (6). The RQ drops below 1 after 29 h, indicating depletion of galacturonic acid liberation. Thus, the line for PolyGalA_calc_ in Fig. 6c is dotted from that time point on. The mean values of biological duplicates are shown for better clarity. Culture conditions: modified Verduyn medium, 0.1 M MOPS and 0.1 M MES, initial pH 6.0, 250 mL flask, filling volume 15 mL, shaking frequency 350 rpm, shaking diameter 50 mm, initial OD_600_ = 0.6, temperature 30 °C

As discussed above, exo-polygalacturonase is exclusively produced during the growth on glucose. Elevated glucose concentrations elongate the enzyme production phase and should consequently lead to an increased enzymatic activity. After glucose depletion, during the growth on polygalacturonic acid, an increased enzymatic activity should therefore correlate with a higher plateau of the OTR. To test this assumption, the glucose concentration during cultivations on polygalacturonic acid was varied between 4 and 20 g/L. The corresponding OTR profiles are depicted in Fig. 6a. All cultivations started to grow equally on glucose. After 8.5, 13.5 and 16.0 h, glucose (4, 12 and 20 g/L) was depleted, respectively, as indicated by the drop in the OTR. After a slight increase while growing unlimited on galacturonic acid, a plateau was reached at an OTR of 5.4, 11.9 and 15.4 mmol/L/h for 4, 12 and 20 g/L glucose, respectively. The elevated OTR plateau for higher initial glucose concentrations indicates a higher enzymatic activity as hypothesized above. This is most probably due to increased enzyme concentrations, as the cultivations with higher glucose concentrations experienced a longer enzyme production phase. During glucose consumption, the cells do not only produce enzyme but also biomass. This leads to increasing biomass concentrations at the beginning of the galacturonic acid consumption phase with increased initial glucose concentrations. Balancing the available amount of galacturonic acid with the biomass results in an equal cell-specific galacturonic acid uptake rate, independent of the initial glucose concentration. The OTR represents the sum of the metabolic activity of all cells in the culture. The higher OTR plateau of cultures with an increased initial glucose concentration, thus, originates from a higher biomass concentration. An increase of the initial glucose concentration above 20 g/L would lead to an increased enzymatic activity but not to an increased cell-specific galacturonic acid uptake rate. The higher OTR indicates both, a higher liberation and consumption rate of galacturonic acid. A high overall substrate metabolization rate results in a shorter cultivation time. This can be observed in a shorter duration of the OTR plateau with 10 h for 20 g/L initial glucose or 12.5 h for 12 g/L initial glucose, when compared to 28.5 h for 4 g/L initial glucose. Remarkably, the overall consumed substrate does not differ substantially between the different cultivations (Fig. 6c). As discussed in the previous section, a high fraction of the substrate remained unused as 8 g/L of initially 20 g/L substrate remain in the medium at the end of the cultivation. This is independent of elongated galacturonic acid consumption phases or the enzyme concentration in the media. It underlines the hypothesis of hindered enzymatic galacturonic acid liberation by single heterologous or esterified polymer units in the polygalacturonic acid chain.

**Fig. 7:**
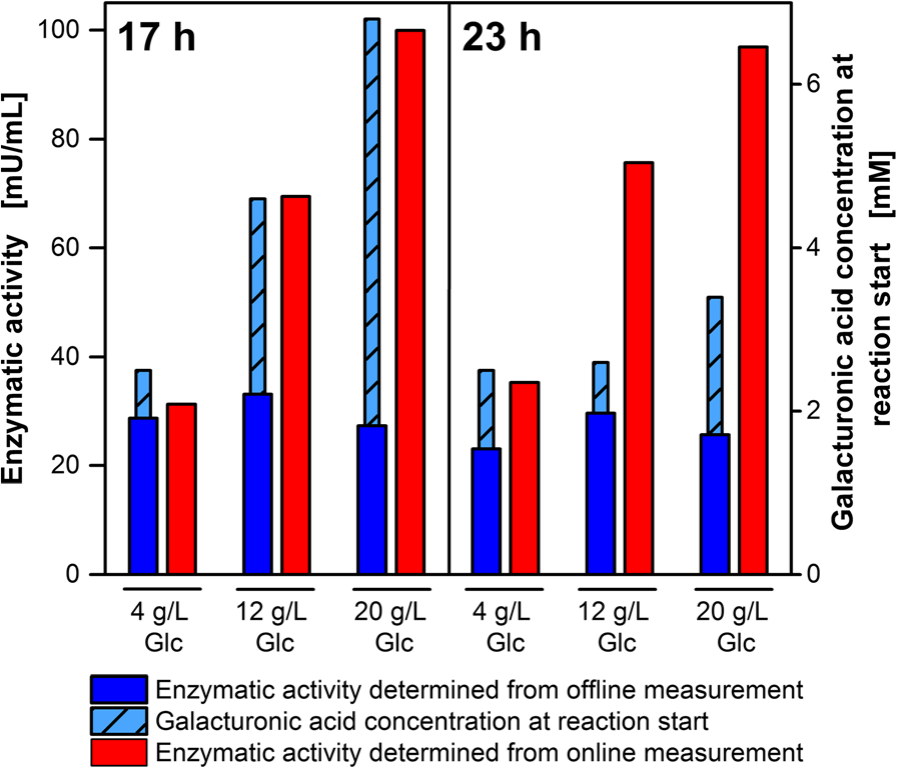
Comparison of enzymatic activity determined from offline (DNS) and online (OTR) measurements. The hydrolytic enzymatic activity on polygalacturonic acid was determined by offline DNS assays (blue bars) or by online determination of the linear substrate consumption shown in Fig. 6 (red bars). Samples of culture supernatant were taken and analysed after cultivation times of 17 and 23 h. For offline assays, the reaction time was 4 h and the polygalacturonic acid concentration 4 g/L. Light blue bars show the galacturonic acid concentration at the beginning of the offline assay

To validate the introduced method for online determination of the enzymatic activity, samples of culture supernatant were drawn after 17 and 23 h of the cultivations on 4, 12 and 20 g/L glucose and 20 g/L polygalacturonic acid shown in Fig. 6. The enzymatic activities determined from offline measurement are clearly different from the online-determined measurements as can be seen in Fig. 7. Offline activities are in the range of 23 to 33 mU/mL for all glucose concentrations at both time points (Fig. 7, blue bars). For the lowest glucose concentration, the online-determined enzymatic activities were in the same order of magnitude (Fig. 7, red bars). However, the online-determined activities for 12 and 20 g/L glucose were up to 4-fold higher than the offline-determined values. This discrepancy between the online and offline enzymatic activity can be explained by examining the enzyme kinetics. The enzyme kinetics for the tested exo-polygalacturonase have been reported to be very susceptible to product inhibition [43]. The enzyme can be competitively inhibited by monomeric galacturonic acid with a kinetic inhibition coefficient of K_I_ = 0.3 mM. As the galacturonic acid concentration at the beginning of the assay reaction is > 2 mM for all offline assays (Fig. 7, hatched blue bars), it is probable that the enzyme is inhibited during the entire offline assay. That would explain the constant outcome for enzymatic activity. The activities determined from online measurement are highly reliable as the product is constantly consumed and the enzyme is not affected by product inhibition. Thus, in case of a product inhibition, the offline determination of enzymatic activities underestimates the real enzymatic activity. Previous reports on product inhibited CAZymes also recommend to set-up consolidated bioprocessing in order to prevent high sugar accumulation by constant consumption of the released sugars [22, 62, 63].

The online determination of enzymatic activities for product inhibited CAZymes provides reliable results, even at low total enzymatic activities. However, two prerequisites have to be fulfilled for the introduced method. First, the expressed enzymes have to liberate fermentable sugar units. If for example endo-enzymes simply degrade the polymeric substrates into shorter oligomers that cannot be further converted, the organism shows no metabolic activity and, thus, fails to grow on the hydrolysis products. An *U. maydis* strain producing the intrinsic endo-polygalacturonase would, thus, show no growth on polygalacturonic acid. Secondly, too high activities of the hydrolytic enzyme would lead to a completely unlimited growth on the liberated fermentable sugar units and, thus, the substrate consumption rate never equals the liberation rate. Under those conditions, no linear decrease of the substrate concentration, as shown in Fig. 6c, could be calculated.

Screening for new CAZyme producers could profit substantially from the introduced methodology. Offline determination of the enzymatic activity would sort out strains that produce product inhibited CAZymes with high activities. Thus, high throughput measurement of the metabolic activity in small scale could help circumvent this pitfall.

### Investigation of enzyme stability by substrate pulsing

**Fig. 8:**
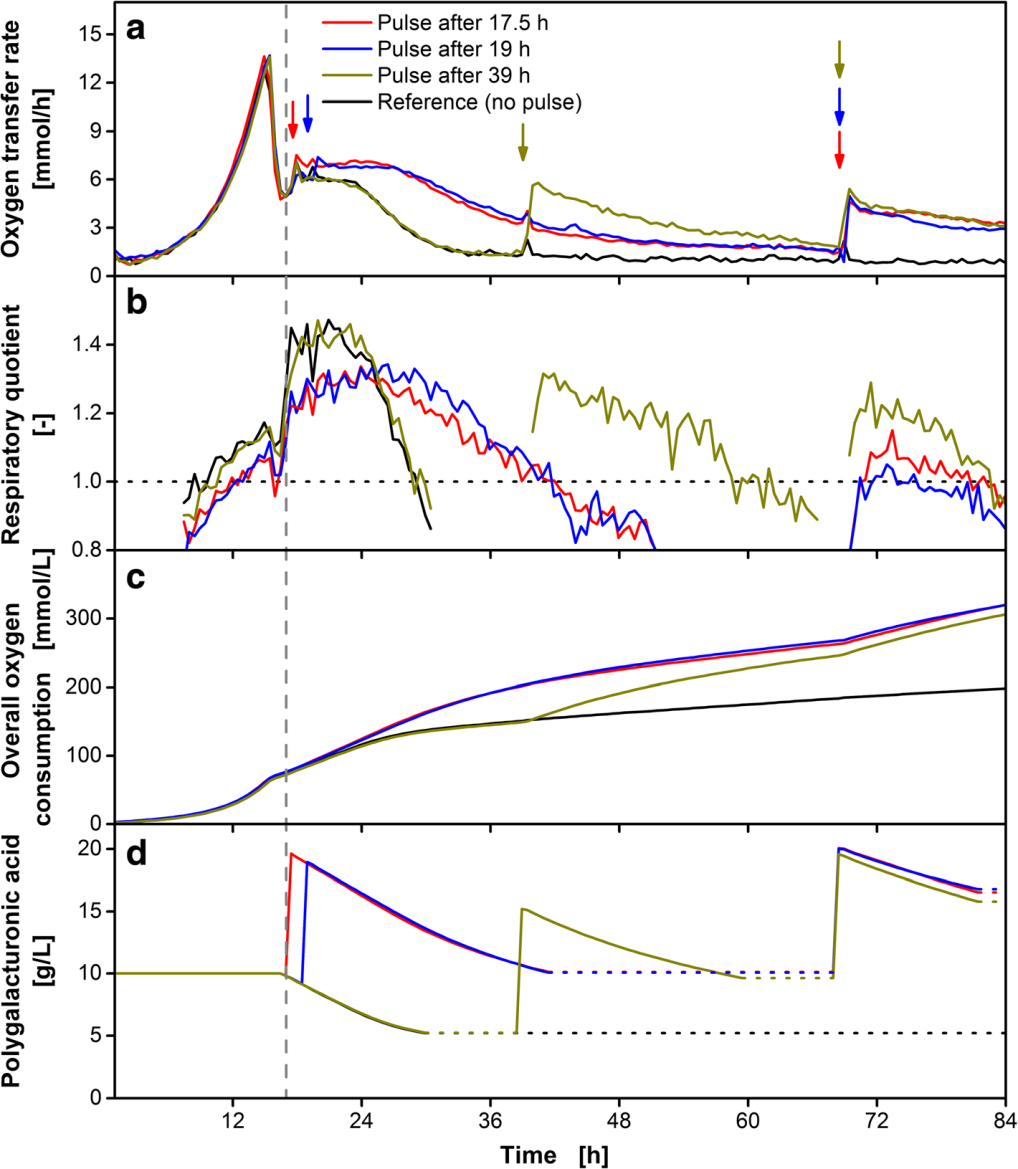
Influence of polygalacturonic acid pulses on the cultivation of *U. maydis* AB33P5ΔR/AtPgaX. The strain was grown on glucose (6.5 g/L) and polygalacturonic acid (10 g/L) as carbon sources. Pulses of polygalacturonic acid were added at indicated time points (arrows). At 68.5 h, all cultures except the reference received a second substrate pulse. The values for the oxygen transfer rate (OTR), the overall oxygen consumption (OT) and the predicted residual polygalacturonic acid concentration (PolyGalA_calc_) refer to the initial filling volume. The vertical dashed line represents the beginning of polygalacturonic acid consumption. **a** Mean value of biological duplicates of the OTR. **b** Mean value of biological duplicates of RQ. The dotted horizontal line represents RQ = 1. For clarity, the RQ is only shown for OTR > 2 mmol/L/h. **c** Mean value of duplicates of the OT. **d** PolyGalA_calc_ according to Eq. (6). The RQ drops below 1 during consumption of polygalacturonic acid, indicating depletion of accessible substrate. Thus, the line for PolyGalA_calc_ in Fig. 8d is dotted during that time. The mean values of biological duplicates are shown for better clarity. Culture conditions: modified Verduyn medium, 0.2 M MOPS, initial pH 6.5, 250 mL flask, initial filling volume 20 mL, shaking frequency 300 rpm, shaking diameter 50 mm, initial OD_600_ = 0.2, temperature 30 °C. The mass of polygalacturonic acid in one pulse equals the initial mass supplemented in the medium. Pulses of 5 mL polygalacturonic acid solution (40 g/L) were added

A consolidated bioprocess for degradation of plant biomass requires highly stable CAZymes. For this study, a haploid *U. maydis* strain, in which five proteases were deleted, was used to prevent degradation of the secreted pectinases. This strain was previously successfully used to produce heterologous single-chain antibodies in *U. maydis* [50]. To prove the enzyme stability of the secreted exo-polygalacturonase and to verify the understanding of the three phases during the growth on polygalacturonic acid discussed previously, polygalacturonic acid was pulsed at different time points as depicted in Fig. 8.

The first time point for a polygalacturonic acid pulse during the unlimited growth phase on galacturonic acid was after 17.5 h (Fig. 8a, red curve). The OTR plateau at 7 mmol/L/h is slightly higher compared to the reference cultivation without pulse (black curve, 6 mmol/L/h). This increase might be associated to more available total polygalacturonic acid substrate. During the plateau phase, the cultures run in parallel until the galacturonic acid liberation rate decreases in the reference cultivation after about 24 h. The OTR plateau of the pulsed culture continues on its level of 7 mmol/L/h until the galacturonic acid liberation rate decreases here as well after about 28 h. The course of the respiratory quotient (Fig. 8b) shows again a clear separation between the growth phases on glucose (< 17 h) and galacturonic acid (> 17 h), as well as the depletion of galacturonic acid. A similar behavior was observed when the pulse was added in the OTR plateau phase after 19 h (Fig. 8a, blue curve). After the pulse, the OTR slightly increased from 6 to 7 mmol/L/h and runs in parallel to the cultivation with the earlier pulse until the end of the experiment.

The reference cultivation (Fig. 8a, black curve) reached a basal level in the OTR after 28 h. Adding a pulse in that cultivation after 39 h (Fig. 8a, olive green curve) results in an immediate increase of the OTR to 6 mmol/L/h, the level of the first plateau. This clearly demonstrates the stability of the enzymes in culture supernatant. Even under starvation of the cells, the enzyme remains functional.

All previously pulsed cultivations were supplemented with a second pulse after 70 h (Fig. 8a, arrows). This pulse resulted in a similar response in all cases. The cultivations reached a similar OTR level. As described for the pulse after 39 h, the OTR started to decrease right after the maximal OTR was reached. The RQ values remained below those values obtained during the first OTR plateau. This indicates a stronger maintenance metabolism due to starvation.

Figure 8c shows the OT. The pulsed cultivations reach comparable final values of 315 mmol/L. The reference cultivation shows a lower final OT of 200 mmol/L. The method for prediction of the residual substrate concentration was applied to compare the overall consumption of the supplemented polygalacturonic (Fig. 8). After the polygalacturonic acid consumption, the reference culture consumed 50% of the supplemented polygalacturonic acid. All pulsed cultivations also ended up at 50% substrate consumption after 60 h and even after consumption of the second substrate pulse (84 h). Thus, even under different cultivation conditions, the percentage of convertible polygalacturonic acid remains constant. The sharp increase in the OTR after the pulses at 39 and 70 h indicates that the presented cultivation system is tolerant towards short starvation times and the secreted exo-polygalacturonase is neither degraded nor inhibited by secreted metabolites of the cells or by other media components.

In future, further efforts have to be made to increase the overall consumption rate of polygalacturonic acid. This might be achieved by implementation of other pectinases. For example, an endo-polygalacturonase would be beneficial which can cleave polygalacturonic acid chains with esterified residues at the non-reducing ends that cannot be hydrolyzed by exo-polygalacturonases. By an intra-chain cleavage, new active sites for the exo-polygalacturonase are provided. With such two-enzyme system, more complex substrates might be addressed, like pectin or pectin-rich biomass waste-streams.

In general, there are two options to achieve the simultaneous production of multiple different CAZymes: The *U. maydis* AB33P5ΔR/AtPgaX strain applied within this study could be modified to overexpress multiple other enzymes. This strategy could be inefficient because too many simultaneously overexpressed enzymes could decrease the production rate for each single enzyme. A different and more promising attempt is to generate novel strains, each overexpressing a different CAZyme. The latter could be cultivated together in a mixed culture.

## Conclusion

In this study, the metabolic activity of *U. maydis* growing on galacturonic acid, the main component of pectin, was characterized by online monitoring tools. The growth behavior was reproducible and enabled closed carbon balancing for the growth on glucose and galacturonic acid. The overall consumed oxygen was successfully correlated with the consumed amount of substrate. A method for the prediction of the residual substrate concentration was developed. The production of the heterologous exo-polygalacturonase PgaX in *U. maydis* activated this strain to grow on polygalacturonic acid. The developed method was applied to calculate the enzymatic activity during the growth of this activated *U. maydis* strain on polygalacturonic acid. A comparison with offline determined enzymatic activities showed that online measurements prevented an underestimation of the enzymatic activity due to inhibition by accumulated product. Thus, the newly introduced method for online determination of the enzymatic activity is highly favorable in case of product inhibited hydrolytic enzymes. Finally, the stability of secreted enzymes was proven over the entire course of fermentation by pulsing substrate at distinct times of the cultivation.

Future research aims to increase the complexity of digestible substrate. For growth on pectin, sugar beet pulp, or other pectin-rich waste streams, a high number of different enzymes such as hydrolases or monooxygenases is required [64]. The presented methodology to characterize the enzyme activity of the investigated strain and to determine the residual substrate concentration could thus be transferred to, e.g., industrial production hosts. The number of CAZymes could alternatively be increased by mixed culture fermentations. This approach seems promising as it mimics nature in terms of microbial cooperation for degradation of plant biomass [65, 66].

## Methods

### Microorganisms

The strains used in this study are specified in Table 2. The genetic modification of *U. maydis* AB33P5ΔR was performed by homologous recombination of a linearized plasmid containing the codon-optimized exo-polygalacturonase gene *pgaX* originating from *A. tubingensis* under control of the constitutive strong promoter P_*oma*_ [20, 42, 43, 50, 67]. The construct was inserted in the *ip* locus mediating carboxin (Cbx) resistance [17].

**Table 2:** List of applied microorganisms and their origin

Strain number	Description	Vector used for modification	Resistance	Progenitor	Manipulated Locus	References
UMa1391	AB33P5ΔR		Phleo	AB33		[50]
UMa2106	AB33P5ΔR/AtPgaX	pUMa3108	Phleo, Cbx	UMa1391	*ip*	(Stoffels, Müller, *et al.*, unpublished data)

### Cultivation medium and conditions

*U. maydis* strains were cultivated in 250 mL shake flasks with a filling volume of 20 mL (shaking diameter 50 mm, shaking frequency 300 rpm, T = 30 °C). All cultivations were performed in modified Verduyn medium [68]. The exact composition was 4 g/L NH_4_Cl, 0.5 g/L KH_2_PO_4_, 0.2 g/L MgSO_4_ ∙ 7 H_2_O, 0.01 g/L FeSO_4_ ∙ 7 H_2_O and 1 mL/L trace element solution. The trace element solution includes 15 g/L ethylenediaminetetraacetic acid (EDTA), 4.5 g/L ZnSO_4_ ∙ 7 H_2_O, 0.84 g/L MnCl_2_, 0.3 g/L CoCl_2_ ∙ 6 H_2_O, 0.3 g/L CuSO_4_ ∙ 5 H_2_O, 0.4 g/L Na_2_MoO_4_ ∙ 2 H_2_O, 4.5 g/L CaCl_2_ ∙ 2 H_2_O, 3 g/L FeSO_4_ ∙ 7 H_2_O, 1 g/L B(OH)_3_, 0.1 g/L KI. The medium was buffered either with 39.04 g/L (0.2 M) 2-(N-morpholino)ethanesulfonic acid (MES) or with 41.86 g/L (0.2 M) 3-(N-morpholino)propanesulfonic acid (MOPS), depending on the applied carbon source. The pH of the medium without carbon source and trace element solution was set to 6.5 with NaOH, if not stated otherwise. The corresponding carbon source (glucose, galacturonic acid or polygalacturonic acid) was dissolved separately at higher concentrations (glucose 500 g/L, galacturonic acid 200 g/L, polygalacturonic acid 40 g/L). For acidic substrates, the pH was adjusted with NaOH. Finally, the dissolved carbon source was added to the medium at varying concentrations. Galacturonic acid was purchased from Sigma-Aldrich (St. Louis, USA). Polygalacturonic acid was purchased form Carl Roth (Karlsruhe, Germany) at a purity of ≥ 85%. All other chemicals were of analytical grade. Glucose, galacturonic acid, FeSO_4_ and buffer solutions were sterile-filtered using 0.2 μm cut-off filters (Acrodisc 32 mm, Pall, Dreieich, Germany). The other solutions were separately autoclaved (121°C, 20 min).

### Cultivation with online monitoring and offline sampling

All cultivations were performed in 250 mL shake flasks, modified for usage in an in-house built RAMOS device [44, 45]. Commercial versions of the device are available from Kühner AG (Birsfelden, Switzerland) or HiTec Zang GmbH (Herzogenrath, Germany). The flasks were filled with 20 mL medium and shaken with a shaking frequency of 300 rpm at a shaking diameter of 50 mm.

For precultures, Verduyn medium with 20 g/L glucose was inoculated with 500 μL cell suspension from a cryostock culture. The cells were grown 16 to 24 h until the carbon source was depleted. After determination of the optical density using a spectrophotometer (Genesys 20, Thermo Fischer Scientific, Waltham, USA), the required volume of culture broth was harvested by centrifugation and the main cultures were inoculated at a defined OD_600_ between 0.2 and 0.65.

For offline samples, cotton plug-sealed shake flasks were cultivated in parallel to the RAMOS flasks under identical cultivation conditions using identical inocula.

### Offline analysis

#### Optical density

Biomass during cultivation in non-turbid media (without polygalacturonic acid) was determined by measuring the optical density at 600 nm (OD_600_) with a spectrophotometer using 1.5 mL micro cuvettes (PS, Plastibrand, Roth, Karlsruhe, Germany). If necessary, samples were diluted with 0.9% (w/v) NaCl solution to a final OD_600_ < 0.3. NaCl solution was used as blank. Each sample was measured in duplicates.

#### pH value

The pH value of unfiltered culture broth was measured at room temperature using a pH-meter (HI2211 pH, Hanna Instruments, Vöhringen, Germany) equipped with a pH-electrode (InLab Easy pH, Mettler-Toledo, Giessen, Germany).

#### Ammonia

The ammonium concentration of culture supernatant was determined after dilution to appropriate concentrations using the Spectroquant test kit (N° 114544, Merck KGaA, Darmstadt, Germany). The samples were measured using a photometer (Nova 60, Merck KGaA, Darmstadt, Germany)

#### Quantification of carbon sources

Glucose and galacturonic acid concentrations were determined from cell-free supernatant by HPLC. After sterile filtration with 0.2 μm filters (Rotilabo syringe filters Mini-Tip cellulose acetate membrane, N° PP52.1, Carl Roth, Karlsruhe, Germany), the samples were separated in the HPLC (Prominence, Shimadzu AG, Kyoto, Japan) using an organic acid resin column (250 x 8 mm, CS-Chromatographie Service GmbH, Langerwehe, Germany). The separation was achieved with 1 mM H_2_SO_4_ at 75°C at a flow rate of 0.8 mL/min. The column was coupled to a refractometer (Shodex RI-101, Showa Denk Europe, Munich, Germany) and the data was analyzed by the software Chromeleon 6.2 (Dionex, Germering, Germany).

#### Cell dry weight

For determination of the cell dry weight, 2 mL culture broth were collected in a dry reaction tube, centrifuged and the supernatant was discarded. After drying the cell pellet for 48 h at 80 °C, it was weighted using an analytical balance (LA 254i, VWR International GmbH, Darmstadt, Germany). All samples were measured as technical triplicates.

#### Enzymatic activity via reducing sugar assay (DNS assay)

Culture broth was collected, centrifuged, and subsequently used for offline determination of the enzymatic activity. 4 g/L polygalacturonic acid solution in 0.1 M MES buffer, pH 6.0 was used as substrate. The assay was performed in one 2 mL reaction tube per time point incubated in a thermoshaker at 30°C and 800 rpm over a period of 24 h. At each sampling point, one reaction tube was heated up to 95 °C for 5 min to inactivate the enzyme. After the last sampling point, the concentration of reducing groups was determined by 3,5-dinitrosalicylic acid (DNS) assay [69]. All samples were mixed with DNS solution (10 g/L DNS, 404 g/L potassium sodium tartrate tetrahydrate, 16 g/L NaOH) at equal volumes. The colorimetric reaction was carried out for 10 min in a thermoshaker at 95 °C and 800 rpm. Finally, the samples were cooled on ice and transferred to a 96-well microplate with a filling volume of 300 μL per well. All samples were measured in technical triplicates. The absorption at 540 nm (A_540_) was measured in a plate reader (Synergy 4, Biotek, Winooski, USA). As internal control for turbid samples, the A_630_ was measured as well as described previously [70]. The final DNS-based absorbance was determined as A_540_-A_630_. Calibration curves were prepared using galacturonic acid at concentrations between 0 and 20 mM. An exemplary calibration curve is shown in Additional file 6. The enzymatic activity was calculated from the increase of reducing groups during the first 4 h. The monomeric galacturonic acid concentration at the beginning of the reaction was determined from the measured reducing groups at the reaction start.

## Additional files

Additional file 1: Comparison of *U. maydis* AB33P5ΔR cultivation in modified Verduyn medium with and without vitamin supplementation. The strain was grown on glucose (4 g/L) and galacturonic acid (20 g/L) as carbon sources. Biological duplicates of oxygen transfer rate. Culture conditions: modified Verduyn medium, 0.1 M MOPS, pH 6.5, 250 mL flask, filling volume 20 mL, shaking frequency 300 rpm, shaking diameter 50 mm, initial OD_600_ = 0.2, T = 30 °C. (TIF 145 kb)

Additional file 2: Control cultivation of *U. maydis* AB33P5ΔR/AtPgaX on galacturonic acid without glucose as starter carbon source. The strain was grown on 20 g/L galacturonic acid as carbon source. Oxygen transfer rate over time for different inoculation densities between OD_600_ = 0.2 and 2.0. Culture conditions: modified Verduyn medium, 0.1 M MOPS, initial pH 6.5, 250 mL flask, filling volume 20 mL, shaking frequency 300 rpm, shaking diameter 50 mm, temperature 30 °C. (TIF 201 kb)

Additional file 3: Respiratory quotient during the cultivation of *U. maydis* AB33P5ΔR on galacturonic acid (same cultivation as shown in Fig. 2). The strain was grown on glucose (4 g/L) and galacturonic acid (20 g/L) as carbon sources. Vertical dashed lines indicate depletion times for glucose (11.5 h) and galacturonic acid (43 h). **a** Biological duplicates of oxygen transfer rate and carbon dioxide transfer rate. **b** Biological duplicates of respiratory quotient. Horizontal dotted line represents RQ = 1. Culture conditions: modified Verduyn medium, 0.2 M MOPS, pH 6.5, 250 mL flask, filling volume 20 mL, shaking frequency 300 rpm, shaking diameter 50 mm, initial OD_600_ = 0.2, T = 30 °C. (TIF 237 kb)

Additional file 4: Development of the pH value during the cultivation of *U. maydis* AB33P5ΔR/AtPgaX on galacturonic acid (same cultivation as shown in Fig. 3). The medium was supplemented with glucose (4 g/L) and varying amounts of galacturonic acid (GalA_0_) and buffer. 0, 4.5, 8.8, 12.8, 16.9, 21.7, 26.1 and 34.6 g/L galacturonic acid medium was supplemented with 0.1, 0.1, 0.1, 0.15, 0.2, 0.25, 0.3 and 0.4 M MOPS respectively. **a** Oxygen transfer rate. **b** pH value determined from shake flasks cultured in parallel. Culture conditions: modified Verduyn medium, initial pH 6.5, 250 mL flask, filling volume 20 mL, shaking frequency 300 rpm, shaking diameter 50 mm, initial OD_600_ = 0.65, T = 30 °C. (TIF 315 kb)

Additional file 5: Control cultivation of *U. maydis* AB33P5ΔR/AtPgaX on glucose and polygalacturonic acid. The strain was grown on 20 g/L glucose or 20 g/L polygalacturonic acid as single carbon source. **a** Oxygen transfer rate. **b** Respiratory quotient. The dotted horizontal line represents RQ = 1. For clarity reasons, the RQ is only shown for OTR > 2 mmol/L/h. Culture conditions: modified Verduyn medium, 0.1 M MOPS and 0.1 M MES, initial pH 6.0, 250 mL flask, filling volume 15 mL, shaking frequency 300 rpm, shaking diameter 50 mm, initial OD_600_ = 0.6, temperature 30 °C. (TIF 198 kb)

Additional file 6: Exemplary calibration curve for offline DNS assay on reducing groups. Samples of 2.5 to 20 mM galacturonic acid were incubated with DNS reagent as described in the materials and methods section. Absorption at 540 and 630 nm was measured in triplicates in a microtiter plate using a plate reader. Error bars represent standard deviation of technical triplicates. The calibration factor and coefficient of determination are specified in the plot. (TIF 116 kb)

## Abbreviations

*A. tubingensis*: *Aspergillus tubingensis*
CAZyme: Carbohydrate-active enzyme
CTR: Carbon dioxide transfer rate mmol/L/h
DNS: 3,5-dinitrosalicylic acid
GalA: Galacturonic acid g/L
MES: 2-(N-morpholino)ethanesulfonic acid mol/L
MOPS: 3-(N-morpholino)propanesulfonic acid mol/L
OT: Overall oxygen consumption mmol/L
OTR: Oxygen transfer rate mmol/L/h
PolyGalA: Polygalacturonic acid g/L
RAMOS: Respiration activity monitoring System
RQ: Respiratory quotient
*T. reesei*: *Trichioderma reesei*
*U. maydis*: *Ustilago maydis*
